# Prediction of response to systemic treatment by kinetics of circulating tumor DNA in metastatic pancreatic cancer

**DOI:** 10.3389/fonc.2022.902177

**Published:** 2022-08-30

**Authors:** Patrick Kirchweger, Alexander Kupferthaler, Jonathan Burghofer, Gerald Webersinke, Emina Jukic, Simon Schwendinger, Helwig Wundsam, Matthias Biebl, Andreas Petzer, Holger Rumpold

**Affiliations:** ^1^ Gastrointestinal Cancer Center, Ordensklinikum Linz, Linz, Austria; ^2^ Department of Surgery, Ordensklinikum Linz, Linz, Austria; ^3^ Medical Faculty, Johannes Kepler University Linz, Linz, Austria; ^4^ Department of Diagnostic and Interventional Radiology, Ordensklinikum Linz, Linz, Austria; ^5^ Laboratory for Molecular Genetic Diagnostics, Ordensklinikum Linz, Linz, Austria; ^6^ Institute of Human Genetics, Medical University of Innsbruck, Innsbruck, Austria; ^7^ Department of Internal Medicine I for Hematology with Stem Cell Transplantation, Hemostaseology and Medical Oncology, Ordensklinikum Linz, Linz, Austria

**Keywords:** metastatic pancreatic cancer, predictive marker, response to systemic treatment, liquid biopsy, circulating tumor DNA

## Abstract

**Introduction:**

Pretherapeutic detectable circulating tumor DNA (ctDNA) represents a promising prognostic biomarker for predicting relapse and overall survival in patients with metastatic pancreatic cancer. However, the prognostic value of ctDNA dynamics during treatment has not been studied thus far. We aimed to investigate the correlation between the change of ctDNA levels and response to treatment in patients treated by systemic therapy.

**Material and methods:**

CtDNA detection using liquid biopsy (droplet digital PCR (ddPCR) utilizing *KRAS* G12/13 and, if negative, Q61 commercial test kits) was prospectively performed on patients with stage IV pancreatic cancer i) prior to initiation of systemic chemotherapy and ii) serially every 2 weeks until restaging. Detection rates, levels of ctDNA, and the course of the relative ctDNA change (ctDNA kinetics) were correlated to treatment response and clinical outcome.

**Results:**

The detection rate at baseline was 64.3% (45/70), and complete serial measurement records were available for 32 ctDNA-positive patients. Reduction of ctDNA levels below 57.9% of its baseline value at week 2 after treatment initiation was significantly predictive of response to treatment (area under the curve (AUC) = 0.918, sensitivity 91.67%, and specificity 100%) and was associated with prolonged overall survival (OS) (5.7 vs. 11.4 months, p = 0.006) and progression-free survival (PFS) (2.5 vs. 7.7 months, p < 0.000) regardless of treatment line. Pretherapeutic ctDNA detection was independently associated with worse OS in patients receiving a first-line regimen (7 vs. 11.3 months, p = 0.046) and regardless of treatment line (11.4 vs. 15.9 months, p = 0.045) as well as worse PFS (3.4 vs. 10.8 months, p = 0.018).

**Conclusion:**

The change in magnitude of ctDNA during systemic treatment allows the prediction of treatment response and is associated with both OS and PFS. This finding adds significant clinical potential to the already established prognostic value of ctDNA positivity in metastatic pancreatic cancer.

## Introduction

Pancreatic cancer is the fourth leading cause of cancer death in both men and women (1), and by 2030, it will become the second leading cause globally (2). Therefore, improvement of survival in pancreatic cancer is of major scientific interest (3). Pancreatic ductal adenocarcinoma (PDAC) is the most prevalent neoplastic disease of the pancreas accounting for more than 90% of all pancreatic malignancies (4). Real-world data show 5-year survival rates of 0.5% in patients with metastatic PDAC and median survival not exceeding 1 year despite extensive treatment regimens including FOLFIRINOX and gemcitabine/nab-paclitaxel (GnP) (3, 5). Treatment evaluation using contrast-enhanced computed tomography (CT) is usually performed in 3-month intervals, revealing progressive disease (PD) in at least 23% of patients receiving a modified FOLFIRINOX regimen (6). Thus, at least one-quarter of patients without a treatment response have to tolerate unnecessary grade 3/4 toxicity (i.e., 23% neutropenia, 5% febrile neutropenia, 12% fatigue, 9% nausea, and 10% diarrhea) until radiological detection (6). Therefore, biomarkers for reliable and early treatment evaluation are needed to potentially prevent unnecessary toxicity. The only blood-based biomarker recommended for clinical application by the National Comprehensive Cancer Network is the plasma protein-derived carbohydrate antigen 19-9 (CA19-9) (3, 7). However, CA19-9’s value in palliative chemotherapy is not uncontroversial, as its values can be substantially disturbed through, for example, cholangitis or other inflammations, which are not uncommon in metastatic pancreatic cancer (3). However, previous studies have demonstrated that CA19-9 is able to distinguish between different mortality risks at baseline, and increased values after 6–8 weeks indicate lower survival rates; this implies indirect early treatment failure, whereas stabilization or high response of this biomarker did not (8). However, this surrogate seems to be capped at 2 months from treatment initiation (9), as changes within 1 month of chemotherapy have not shown to predict outcome (10). In recent years, circulating tumor DNA (ctDNA) has been established as a particularly promising prognostic biomarker with superiority in terms of specificity (83%), sensitivity (100%), and lead time (estimated to be 1–2 months) of relapse prediction compared to current gold standard imaging or CA19-9 (3, 11–13). CtDNA represents tumor burden, as it shows a strong correlation with total tumor volume, especially hepatic lesion volume (14, 15). Moreover, liquid biopsy offers the possibility of minimally invasive, easily reproducible (simple blood collection), and real-time assessment of the continual tumor burden (14, 15). Theoretically, PDAC represents an ideal tumor entity for minimally invasive mutation screening with commercially available test kits suitable for clinical practice without the need for prior histological target definition, as >90% of patients have a *KRAS* mutation in the tissue (14). However, liquid biopsy allows detection rates of only 50%–65% in metastatic PDAC (3, 11, 15). While the prognostic value of ctDNA positivity prior to treatment has been validated for metastatic PDAC (13, 16), there is no established cutoff value for the ctDNA change reflecting an early response to treatment. Thus, we aimed to evaluate if a patient-normalized threshold based on the dynamic change of ctDNA during systemic treatment can predict response to systemic treatment and clinical outcomes in terms of disease-free survival (DFS) and overall survival (OS).

## Material and methods

### Patients

**Figure 1:**
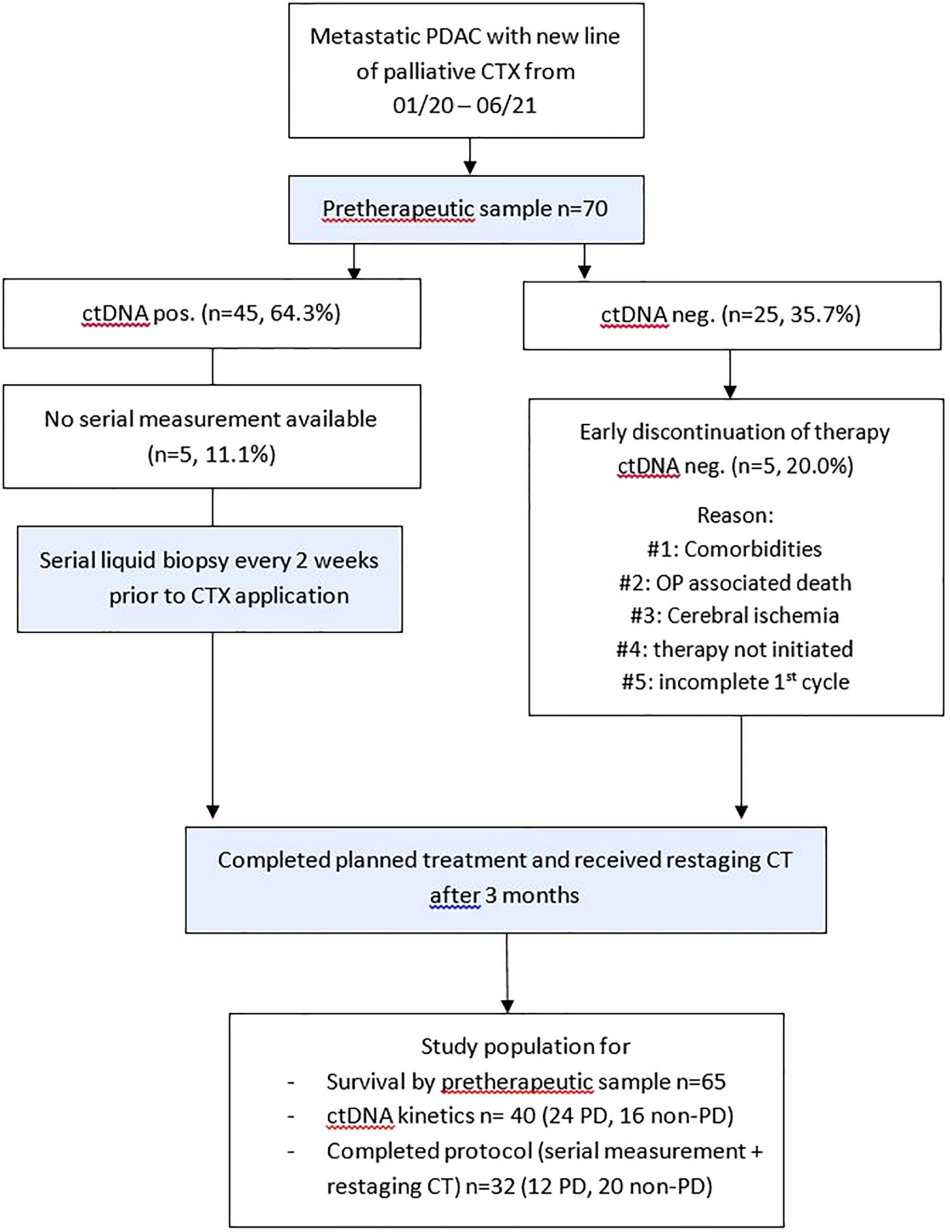
Study flow diagram. Patients with metastatic PDAC and planned application of a new line of palliative chemotherapy were included. CT, computed tomography; ctDNA, circulating tumor DNA; CTX, chemotherapy; neg., negative; non–PD, non–progressive disease (complete response, partial response, stable disease); OP, operation; PD, progressive disease; PDAC, pancreatic ductal adenocarcinoma.

A total of 70 patients receiving palliative chemotherapy in either treatment line for pancreatic cancer at our oncological center between January 2020 and June 2021 were prospectively included in this study. The study flow diagram is provided in **Figure 1**. Study participation did not affect the treatment decisions, which were blinded to study results. The treatment decision was based on local treatment guidelines, which are based on the European Society for Medical Oncology (ESMO) guidelines. Clinical and follow-up data were obtained from the prospective cancer registry of the hospital. Written informed consent was obtained from all study participants. This study was approved by the ethics committee of the hospital (EK 70/90).

### Plasma collection and processing

Pretherapeutic plasma samples for the liquid biopsy analysis were collected from all 70 patients with metastasized pancreatic cancer the day before treatment initiation and every 2 weeks thereafter until restaging before chemotherapy infusion (median of six samples (interquartile range (IQR) 4–8) in ctDNA-positive patients and a median of seven samples (IQR 6–8) in ctDNA-negative patients, p = 0.088). A total of 28.5 ml of blood was drawn using cell-free DNA collection tubes (Roche, Basel, Switzerland). After centrifugation at 200 g for 10 min, the supernatant was transferred into new 15-ml tubes (Sarstedt, Nümbrecht, Germany). Another centrifugation at 1,500 *g* for 10 min resulted in 10 ml of plasma, which was then again transferred into a new 15-ml tube. Storage was at −20°C until the DNA was prepared.

### Processing of circulating cell-free DNA

Circulating cell-free DNA (cfDNA) preparation was performed with 10 ml of plasma on the Chemagic 360 (PerkinElmer, Waltham, MA, USA) using the kit CMG-1304 (PerkinElmer, Waltham, MA, USA) according to the manufacturer’s instructions. DNA elution was prepared with a 70 µl elution buffer CMG-844 (PerkinElmer, Waltham, MA, USA) resulting in a DNA volume of 40~50 µl (natural loss as residual liquid in the beads). Quantification was performed using the Quantus fluorometer (Promega, Madison, WI, USA). Samples were stored at 4°C until the next use.

### Droplet digital PCR

The QX200™ Droplet Digital™ PCR System from Bio-Rad (Bio-Rad Laboratories, Hercules, CA, USA) was used to screen for *KRAS* alterations. All samples were screened for variants in *KRAS* G12/G13 utilizing a commercial multiplex screening kit (Bio-Rad Laboratories ddPCR NRAS G12/G13 Screening Kit, Article No.: 12001627). *KRAS* G12/13-negative samples were then further screened for alterations in *KRAS* Q61 using the corresponding kit from the same manufacturer (Bio-Rad Laboratories ddPCR KRAS Q61 Screening Kit, Article No.: 12001626). In serially evaluated patients with positive pretherapeutic samples, all collected samples were analyzed using droplet digital PCR (ddPCR). When the pretherapeutic sample was negative for KRAS G12/13 and Q61 screening kits, the samples were further analyzed from the last time point and the time point with the highest cfDNA concentration (a total of 3× ddPCR analyses per ctDNA-negative patient).

Analyses were performed adhering to the provider’s publicly available instructions. Two reactions (20 µl each) were used for every sample being analyzed. Whenever possible, 5 ng of cfDNA was analyzed per reaction; otherwise, the maximum possible volume of cfDNA was utilized. QuantaSoft™ Analysis Pro software (version 1.0.596) was used for data analysis. Positivity was defined with a threshold of three mutant droplets.

### Radiological analysis

All radiological analyses, including staging, restaging, and volumetric analysis, were performed by the same specialized radiologist who was blinded to treatment, laboratory, or outcome results. Evaluated images were contrast-enhanced dual-energy CT scans in the arterial and portal venous phases prior to treatment initiation and at restaging using Syngo.via (Siemens Healthcare, Forchheim, Germany). Semiautomated lesion detection of the respective organ was used in MM Oncology Workflow mode. If the software failed to define the lesion margin properly, a manual correction was applied. All pathological solid organ lesions were included in the calculations of the total tumor volume and sum of the largest tumor diameter (SLD) assessment regardless of their size. Adhering to Response Evaluation Criteria in Solid Tumors (RECIST) criteria, pathological lymph nodes were only included when the short axis exceeded 10 mm in size (17).

### Statistics

All statistical analyses were performed using the software R version 4.1.2. Survival analyses, Kaplan–Meier curves, log-rank tests, and estimation of hazard ratios were performed by the functions survfit, survdiff, and coxph from the R survival package. Visualization of survival data was performed using SPSS 26.0. The median follow-up time was calculated by the reverse Kaplan–Meier method. Progression-free survival (PFS) was defined as the time from the start of chemotherapy to PD or death. Overall survival (OS) was defined as the time from treatment initiation to death. CtDNA kinetics as an individual’s change of mutant allele frequency (MAF) over time was defined as the quotients of MAF after 2 weeks (ctDNA_ratio_2wk), 4 weeks (ctDNA_ratio_4wk), and restaging (ctDNA_ratio_restaging) divided by its baseline value. Area under the curve (AUC) analyses were run by the function roc (R package pROC). To identify the best cutoff in AUC analysis, the point closest to the top-left part of the plot (best combination of sensitivity and specificity) was chosen. The Mann–Whitney U-test was applied for continuous (i.e., MAF) and discrete (i.e., sex) variables in group comparisons (by ctDNA detection). A two-sided level of significance of 5% and 95% confidence intervals were used.

## Results

### Patient characteristics

The majority of the 70 included patients were treatment naive (71.4%). Patients did not differ in the treatment regimen applied in terms of age, sex, Eastern Cooperative Oncology Group Performance Status (ECOG-PS), discontinuation of therapy rate, median treatment time, median time until restaging, or synchronous/metachronous dissemination when comparing for ctDNA detectability. However, higher treatment line (p = 0.023), the presence of liver metastasis (p = 0.001), larger liver metastasis volume (p = 0.014) or total tumor volume (p = 0.016), and higher CA19-9 levels (p = 0.000) were favorable for ctDNA detection. A larger pretherapeutic SLD was favorable for ctDNA positivity, although it did not reach statistical significance (p = 0.078). *KRAS* G12/13 screening detected mutations in 55.7% (n = 39/70), and further testing with *KRAS* Q61 screening in formerly negative samples revealed mutations in a further 8.6% (n = 6/31, 19.4%). Thus, the detection rate in pretherapeutic liquid biopsy samples was 64.3% (n = 45/70). The mutational distribution pattern using liquid biopsy in this study is given in **Supplementary Figure 1**. Median MAF over the whole study population was 1.6% (IQR 0.3–5.1). An overview of the exact amount of extracted DNA using liquid biopsy is given in **Supplementary Table**. Restaging was performed at a median of 12.1 weeks (IQR 9.6–13.0), and the study population did not differ regarding the time until restaging (p = 0.264), the proportion of progressive disease (p = 0.653), or the time of treatment exposition (p = 0.741) detected between the two groups. Detailed patient characteristics are given in **Table 1**.

**Table 1:** Patient characteristics.

Patient characteristics	Number of patients (%)Age, CA19–9, SLD, volumes, MAF: median (IQR)						
	Overall(n = 70)		CtDNA+(n = 45)		CtDNA−(n = 25)		p
Age (years)	66	(58–73)	66	(59–73)	67	(60–73)	0.825
Male sex	43	(61.4)	28	(62.2)	15	(60.0)	0.856
ECOG–PS							
0	48	(67.4)	33	(80.5)	15	(60.0)	0.114
1	13	(23.9)	5	(12.2)	8	(32.0)	0.041*
≥2	4	(8.7)	3	(7.3)	1	(4.0)	0.613
Treatment line							
1	50	(71.4)	28	(62.2)	22	(88.0)	0.023*
≥2	20	(28.6)	17	(37.8)	3	(12.0)	
Treatment regimen							
FOLFIRINOX	13	(18.6)	8	(17.8)	5	(20.0)	0.682
5FU/Naliri	12	(17.1)	8	(17.8)	4	(16.0)	0.992
GnP	38	(54.3)	24	(53.3)	14	(56.0)	0.478
Others	7	(10.0)	5	(11.1)	2	(8.0)	0.680
Discontinuation of therapy	10	(14.3)	5	(11.1)	5	(20.0)	0.236
Time of treatment	10.8	(5.1–12.6)	9.6	(4.7–12.5)	11.0	(5.1–12.7)	0.741
Time until restaging	12.1	(9.6–13.0)	12.2	(9.7–13.1)	11.4	(9–12.7)	0.264
Site of metastasis							
Liver	53	(75.7)	40	(88.9)	13	(52.0)	0.001*
Lung	20	(28.6)	12	(26.7)	8	(32.0)	0.638
Lymph nodes	11	(15.7)	7	(15.6)	4	(16.0)	0.961
Peritoneum	15	(21.4)	8	(17.8)	7	(28.0)	0.321
Metachronous dissemination	31	(44.3)	18	(40.0)	13	(52.0)	0.336
SLD (mm)	44	(30.8–85.4)	49.9	(34–103.4)	42.3	(17.6–57.7)	0.078
Total tumor volume (mm^3^)	27.9	(7.8–98.7)	30	(11–139.4)	11.7	(4.9–58.5)	0.016*
Liver metastasis volume (mm^3^)	2.64	(0–23.6)	10.9	(0–46.7)	0	(0–2.9)	0.014*
CA19–9 (kU/L)	1,014	(252–5,608)	3,074	(983–32,499)	286	(48–650)	0.000*
MAF (%)	1.6	(0.3–5.1)	1.6	(0.3–5.1)			
KRAS G12/13	39	(55.7)	39	(86.7)			
KRAS Q61	6	(8.6)	6	(13.3)			

Treatment time and time until restaging in weeks.

ECOG–PS, Eastern Cooperative Oncology Group Performance Status; Naliri, nanoliposomal irinotecan; GnP, gemcitabine nab–paclitaxel; SLD, sum of the largest tumor diameter; PD, progressive disease; MAF, mutant allele frequency (%); KRAS, Kirsten rat sarcoma.

*Statistically significant.

### Correlation of circulating tumor DNA dynamics and response to treatment

Early ctDNA dynamics, namely, the ratio of MAF at baseline to the patient’s value after 2 weeks of treatment, and response to treatment were available for 32 patients (PD n = 12, non-PD n = 20).

**Figure 2:**
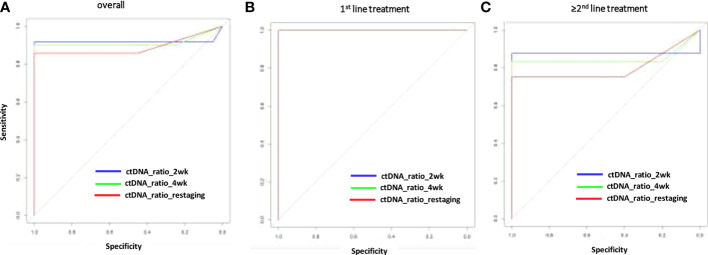
Sensitivity and specificity (ROC analysis) for response prediction by ctDNA reduction during systemic treatment. Relative ctDNA reductions at 2 or 4 weeks or restaging for the overall population **(A)**, the first–line treatment population **(B)** for patients with >1 treatment line **(C)**. AUC, area under the curve; ctDNA, circulating tumor DNA; MAF, mutant allele frequency; ROC, receiver operating characteristics.

**Figure 3:**
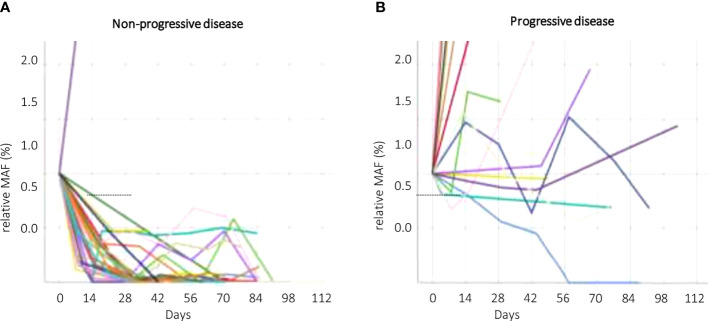
CtDNA kinetics according to response groups. Non–progressive disease (non–PD, **A**) and progressive disease (PD, **B**). Different patients (ctDNA kinetics) are symbolized by different colors. ctDNA, circulating tumor DNA; non–PD, non–progressive disease (complete response, partial response, stable disease); MAF, mutant allele frequency; PD, progressive disease.

The change of MAF over time was significantly different for the PD and non-PD groups at restaging (p < 0.000) and 4 weeks thereafter (p < 0.000), as well as after 2 weeks of treatment initiation (p < 0.000). Receiver operating characteristics (ROC) analysis revealed that a decrease of ctDNA levels within the first 2 weeks of antineoplastic treatment at below 57.9% of the baseline value predicted response to treatment (non-PD group). A ctDNA level above 57.9% at the same time point predicted PD. Specificity for this cutoff value after 2 weeks of treatment is 100% and sensitivity was 91.67% (AUC = 0.918) in the whole population (**Figure 2A**). The predictive value was similar in patients receiving first-line treatment or higher (**Figures 2B, C**). Thus, response prediction utilizing ctDNA kinetics was possible at a median of 14 days (IQR 8–15). The median time to response assessment by conventional CT scan was 97 days (IQR 75–107). This resulted in a median difference in treatment evaluation time of 78 days (IQR 60–89) and a possible reduction of unevaluated cytotoxic treatment exposed time of 84.8% (IQR 81.58–86.54). An illustration of the relative ctDNA changes for each patient during the treatment period is depicted in **Figure 3**.

### Correlation of circulating tumor DNA dynamics with overall survival and progression-free survival

**Figure 4:**
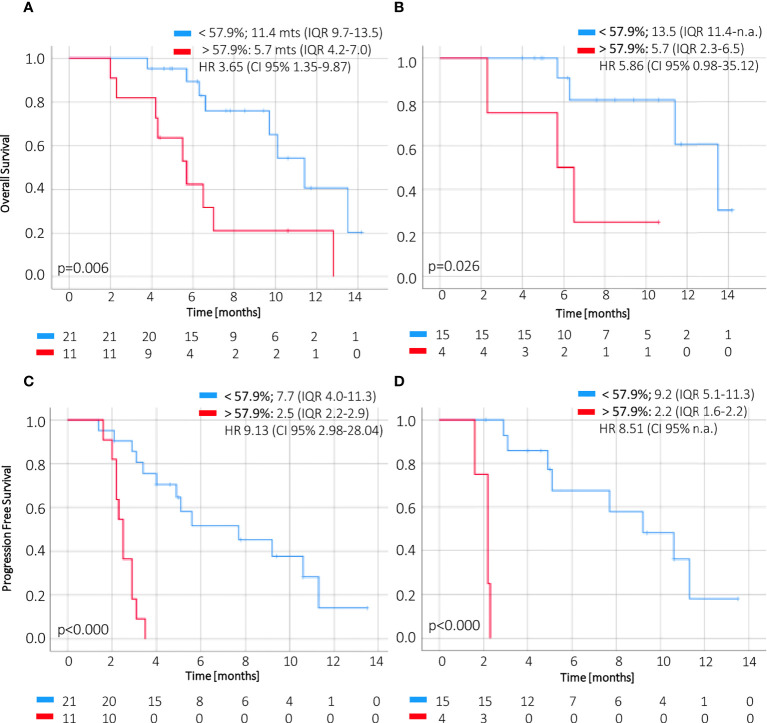
OS and PFS according to magnitude of ctDNA reduction at week 2. The change of ctDNA levels at 2 weeks of treatment below or above 57.9% (i.e., a reduction higher or less than 42.1%) of the baseline value correlates with overall survival **(A, B)** and progression–free survival **(C, D)** in ctDNA–positive patients. CI, confidence interval; HR, hazard ratio; IQR, interquartile range; OS, overall survival; PFS, progression–free survival.

CtDNA dynamics applying a cutoff of 57.9% change at week 2 compared to baseline showed significant association with OS in the first line (5.7 months IQR 2.3–6.5 vs. 13.5 months IQR 11.4–n.r., p = 0.045) and for all treatment lines (5.7 months IQR 4.2–7.0 vs. 11.4 months IQR 9.7–13.5, p = 0.007) (**Figures 4A, B**). Furthermore, this threshold of ctDNA kinetics at week 2 was also significantly related to worse PFS in patients receiving first-line treatment (2.2 months IQR 1.6–2.2 vs. 9.2 months IQR 5.1–11.3) and regardless of treatment line (2.5 months IQR 2.2–2.9 vs. 7.7 months IQR 4.0–11.3) (**Figures 4C, D**).

### Correlation of circulating tumor DNA dynamics with tumor burden

There was a significant correlation between ctDNA levels (i.e., MAF in %) and the respective total tumor volume (R^2^ = 0.504, p = 0.004, **Supplementary Figure 2A**) and liver metastasis volume (R^2^ = 0.543, p = 0.002) at the start of treatment. However, pretherapeutic CA19-9 did not correlate with these volumetric data (R^2^ = 0.042, p = 0.796; R^2^ = 0.160, p = 0.307). SLD did not correlate with ctDNA (R^2^ = 0.254, p = 0.221) nor with CA19-9 (R^2^ = 0.225, p = 0.169). However, dynamic changes of ctDNA (R^2^ = 0.555, p = 0.049) and CA19-9 (R^2^ = 0.720, p < 0.000) between the start of treatment and restaging correlated with the respective change of total tumor (R^2^ = 0.821, p < 0.000) during this period. In contrast to CA19-9, which according to literature may allow early response estimation not before weeks 6–8, ctDNA kinetics as early as week 2 showed strong correlations with the respective continuous change of ctDNA (R^2^ = 0.882, p < 0.000), CA19-9 (R^2^ = 0.889, p < 0.000), and response (p < 0.000) at restaging (**Supplementary Figures 2B–D**).

### Prognostic impact of circulating tumor DNA positivity

Pretherapeutic ctDNA detection was associated with worse OS in patients receiving first-line chemotherapy (11.4 months IQR 7.2–13.5 vs. 15.9 months IQR 7.8–n.r.) and independent of treatment line (7.0 months IQR 2.2–12.8 vs. 11.3 months IQR 7.2–n.r., p = 0.045) (**Supplementary Figures 3A, B**). Similar results were found for PFS, also regardless of treatment line (3.4 months IQR 2.1–9.2 vs. 10.8 months IQR 2.9–13.6), although data did not reach statistical significance in the first-line cohort (5.1 months IQR 2.9–11.3 vs. 10.8 months IQR 3.0–13.6, p = 0.139) due to the short follow-up (**Supplementary Figures 3C, D**). Thus, the OS of patients with a decrease of ctDNA below 57.9% compared to baseline after 2 weeks of treatment was comparable to that of patients with pretherapeutic non-detectable ctDNA.

There was a significant benefit regarding OS (overall cohort: 5.7 (IQR 4.2–7.0) vs. 12.8 (IQR 9.7–13.5) months, p = 0.001; first-line treatment: 5.7 (IQR 5.7–n.r.) vs. 13.5 (IQR 11.4–n.r.) months, p = 0.019) and PFS (overall: 2.9 (IQR 2.2–3.4) vs. 5.6 (IQR 4.0–11.3) months, p < 0.000; first-line treatment: 2.2 (IQR 2.2–2.9) vs. 10.6 (IQR 5.1–11.3) months, p = 0.001) of patients who were pretherapeutic ctDNA positive turning ctDNA negative during chemotherapy regardless of treatment line as compared to those patients who stayed ctDNA positive. Moreover, patients with pretherapeutic ctDNA positivity reached similar OS/PFS when turning ctDNA negative during chemotherapy compared to patients who were ctDNA negative at the start of treatment (**Supplementary Figure 4**).

## Discussion

### Response to treatment prediction by circulating tumor DNA kinetics

Despite previous understanding of the prognostic value of pretherapeutic ctDNA detection, there is limited knowledge until now about definitive thresholds for ctDNA change and its value for early response prediction in pancreatic cancer. In contrast, a threshold for early response to treatment evaluation has already been discussed for disseminated colorectal cancer (10-fold change by Tie et al.) (18). However, in colorectal cancer, the proportion of mutant alleles detectable in patients’ blood is about 10-fold higher compared to patients with pancreatic cancer, which narrows the diagnostic window for ctDNA application in metastatic PDAC. Rates of non-tissue-informed ctDNA detection in metastatic pancreatic cancer vary between 40% and 67% (ddPCR, next-generation sequencing (NGS), and BEAMing) and reach up to 75% in tissue-informed approaches (BEAMing) (3, 19). Recently, published detection rates of 56.8% were found in the largest patient cohort to date investigating ctDNA in mPDAC (n = 255, retrospective) from Prodige 35 and Prodige 37 using ddPCR of two methylated markers (HOXD8 and POU4F1) (13). These findings align with our results of 64.3% (45/70) in patients with metastatic PDAC without prior mutation analysis from tumor tissue. This shows that the use of commercially available *KRAS* G12/13 and Q61 test kits is a promising method for implementation in clinical routine. However, current technologies seem to face a sensitivity problem in pancreatic cancer (especially in less advanced tumor stages) revealing significantly lower ctDNA detection rates as compared to other gastrointestinal malignancies. Thus, recently, several companies (f.e. Inivata, Signatera, Personalis) invented novel analyzing methods (f.e. individual panels driven by tissue–informed NGS or broad–spectrum analysis of alterations in multiple cancer–related genes) that up to now have been tested experimentally to potentially address the improvement of minimal residual disease (MRD) in several tumor entities that could lead to an additional detection of a further 25% of tissue ctDNA–positive patients up to now undetectable in liquid biopsy of pancreatic cancer (20–22). Unfortunately, those applications are not ready for clinical routine application yet, as the turnaround time from designing individual panels from tissue samples to actual liquid biopsy results that allow clinical decision–making is currently up to about 6–8 weeks (22). Nevertheless, more studies on this promising field of research are needed to prospectively evaluate the clinical impact of treatment change using more sensitive approaches.

Regarding a predictive endpoint for response to systemic chemotherapy treatment, a study suggested time until ctDNA normalization (ctDNA of initially positive patients becoming non–detectable during the course of the disease) as a predictive endpoint for treatment response (3). The authors found that patients becoming ctDNA negative at week 4 had a benefit from treatment. Our findings enabled us to define a specific threshold of the relative decrease in ctDNA as a cutoff in patients with metastatic pancreatic cancer. A ctDNA decrease below 57.9% of its baseline value at 2 weeks was predictive for response to treatment with high sensitivity and specificity and superiority to CA 19–9. The current study demonstrates that continuous detection of ctDNA levels during systemic treatment is feasible and could be beneficial for patients by potentially sparing insufficient cytotoxic treatments for 10–12 weeks if compared to treatment reevaluation by CT.

### Prognostic impact of circulating tumor DNA kinetics

In addition to prognostic information of higher MAF values at baseline (i.e., above the median), the dynamic change of MAF during the course of treatment is even more visible in prognosis (3,24). Moreover, the newly established cutoff for ctDNA kinetics at week 2 enables to distinguish very high–risk patients (median OS 5.7 months for above the cutoff vs. 7 months for ctDNA pos.) from patients with similar survival rates as ctDNA–negative patients (median OS 11.4 months for below the cutoff vs. 11.3 months for ctDNA neg.) independently to treatment lines even within the subgroup of ctDNA positives. An even more drastic risk stratification can be applied for PFS (2.5 vs. 7.7 months). Recently, the prognostic impact of the relative change of ctDNA between treatment initiation and restaging has been shown in a small cohort (n = 14) by commercial test kits (16). However, to the best of our knowledge, a cutoff for MAF dynamics with a direct impact on the outcome for patients with metastatic pancreatic cancer has not been described so far. Research shows that CA19–9 is able to distinguish between different mortality risks at baseline and that increased values after 6–8 weeks indicate lower survival rates as indirect early treatment failure, whereas stabilization or high response did not (8). However, this surrogate seems to be capped at 2 months from treatment initiation (9), as changes within 1 month of chemotherapy did not predict outcome (10).

### Prognostic impact of pretherapeutic circulating tumor DNA detection

Detection of pretherapeutic ctDNA has a significant prognostic impact on OS regardless of the complexity and coverage of screening methods mentioned above ranging from 8.2 vs. 12.6 months reported by Pietrasz et al. (p < 0.001) using ddPCR to 16.8 months, versus not reached as reported by Schlick et al. (p = 0.031) using Idylla™ kits (13,17). This is in concordance with our findings of 11.4 vs. 15.9 months in patients receiving first–line treatment (p = 0.046) and 7 vs. 11.3 months independent of treatment line (p = 0.045). However, prognostic information at a single timepoint before treatment initiation of ctDNA positivity compared to negativity (HR 1.62, 95%CI 1.05–2.49, p = 0.029) is similar to the already established and easier assessable biomarker CA19–9 when higher than the median of 1,366 U/ml (HR 1.7, 95%CI 1.17–2.49, p = 0.006) (13).

There was a larger difference in the prognostic impact of ctDNA detection on PFS in our cohort (3.4 vs. 10.8 months) compared to the results published by Pietrasz et al., which surprisingly did not find a clinically relevant difference in 354 patients treated in the first–line setting (5.3 vs. 6.2 months) (13). This might be due to the number of patients in our cohort being smaller and comprising patients treated in the first line and later lines. Further, a significant association between the respective mutated gene locus and PFS could explain the discrepancy between different study results depending on the coverage of the screening method (23). Botrus et al. found a median PFS of 5.8 vs. 12.9 months (*KRAS*), 5.9 vs. 10.9 months (*TP53*), and 3.7 vs. 8.2 months (*CCND2*) (23). Moreover, within patients with *KRAS* mutation, having two or more alterations resulted in a further reduction of PFS to 3.7 months (23).

There was a significant benefit regarding OS and PFS of patients who were pretherapeutic ctDNA positive and turned ctDNA negative during chemotherapy regardless of treatment line compared to those patients who stayed ctDNA positive. Moreover, patients with pretherapeutic ctDNA positivity seem to reach similar OS/PFS when turning ctDNA negative during chemotherapy as compared to patients who are ctDNA negative at the start of treatment (Supplementary 4). However, despite the impact of the prognostic information of ctDNA normalization, the potential actual clinical benefit deriving from this information on the treatment regimen (potential early change of treatment) is superior using the cutoff presented in this study at week 2 after treatment initiation.

### Outlook

Liquid biopsy is a valuable tool to support clinical decision–making in metastatic pancreatic cancer by a real–time display of tumor burden and its change during the disease course. This study increases knowledge about the potential use of continuous evaluation of ctDNA changes to predict a potential clinical benefit from systemic treatment much earlier than conventional CT scanning. This is promising as insufficient treatment may be avoided very early during therapy by, for example, changing or escalating systemic treatment based on missing ctDNA decline. However, systemic treatment might be de–escalated in case of sufficient ctDNA decline. However, large–scale prospective studies focusing on such early adaption of therapy guided by serial liquid biopsy results are needed to validate our data.

### Limitations of the study

Potential evaluation bias of patients at different treatment lines (first–line, second–line, and third–line treatments) may have introduced heterogeneity. However, we addressed this potential bias by analyzing survival data in patients receiving first–line chemotherapy and patients regardless of treatment line independently. However, applied treatment was independent of the study findings, and germline sequencing was not performed in the patients. Thus, in survival analysis, it was not considered whether, for example, patients had a germline pathogenic variant in HRD genes or whether BRCA–mutated patients received platinum–based therapy, which could have led to improved survival, or not.

For the sake of testing a generally available clinically applicable method, using only commercially available KRAS G12/13 and Q61 target assays, we intentionally missed out on evaluating potential TP53, SMAD4, or CDKN2A mutations that would eventually have been detected by NGS, which could have led to significantly higher detection rates. Naturally, if undetectable *via* ctDNA in peripheral blood, it cannot be stated whether the patient is KRAS wild type or just not detectable within the scope of the method without further paired tissue analysis.

Naturally, our findings are limited by the sample size and need to be validated on a large prospective scale.

## Data availability statement

The raw data supporting the conclusions of this article will be made available by the authors, without undue reservation.

## Ethics statement

The studies involving human participants were reviewed and approved by Lokal Ethics Committee of the hospital (EK 70/90). The patients/participants provided their written informed consent to participate in this study.

## Author contributions

PK organized the plasma collection process, drafted the manuscript, created the tables and figures, performed the statistical analysis along with an external department for statistics and mathematics, and performed the corrections, leading to the final manuscript. AK performed the radiological response assessment and volumetric analyses. JB, GW, EJ, and SS performed the DNA processing and interpretation of ddPCR results along with clinicians PK and HR. HW, MB, AP, and HR reviewed the manuscript and assisted in the clinical interpretation of the data. HR had the idea for the project, designed and led the project, was responsible for financing, assisted in statistical analysis, and was the project supervisor in general. All authors contributed to the article and approved the final version of the manuscript.

## Funding

This study was supported by the Vinzenzgruppe Austria, which is the owner of the hospital, the Krebshilfe Oberösterreich, which is a public research funding organisation, and the Johannes Kepler Open Access Publishing Fund to cover the costs of the publication. The funders were not involved in the design or interpretation of the results.

## Conflict of interest

The authors declare that the research was conducted in the absence of any commercial or financial relationships that could be construed as a potential conflict of interest.

## Publisher’s note

All claims expressed in this article are solely those of the authors and do not necessarily represent those of their affiliated organizations, or those of the publisher, the editors and the reviewers. Any product that may be evaluated in this article, or claim that may be made by its manufacturer, is not guaranteed or endorsed by the publisher.

## Supplementary material

Supplementary Figure 1 Correlation of ctDNA and tumor burden. Correlation of MAF at baseline with total tumor volume **(A)**, the dynamic change of MAF from baseline to restaging with the respective CA19–9 change **(B)** and similar relation for MAF change from baseline to 2 weeks after treatment initiation with the change of CA19–9 from baseline to restaging **(C)**. ctDNA as indicator for tumor burden predicting response after 2 weeks of antineoplastic treatment **(D)**. ctDNA, circulating tumor DNA; MAF, mutant allele frequency; nonPD, non–progressive disease; PD, progressive disease; R^2^, Spearman’s rho.

Supplementary Figure 2 Outcome of pretherapeutic ctDNA detection. Pretherapeutic ctDNA detectability correlates with worse OS **(A, B)** and PFS **(C, D)** in first line chemotherapy and regardless of treatment line. HR, hazard ratio; IQR, interquartile range; OS, overall survival; PFS, progression–free survival.

Supplementary Figure 3 Distribution of mutational pattern evaluating peripheral blood.

Supplementary Figure 4 Prognostic impact of ctDNA change during treatment. Patients with pretherapeutic positive ctDNA who turned ctDNA negative during palliative chemotherapy (blue) had significantly improved OS and PFS compared to patients whose ctDNA was positive throughout the course of therapy (red) in the overall cohort **(A, C)** and in patients receiving first line treatment **(B, D)**. Furthermore, it seems that patients turning ctDNA negative during treatment show similar OS and PFS as patients with pretherapeutic negative ctDNA (green). ctDNA, circulating tumor DNA; IQR, interquartile range; OS, overall survival; PFS, progression–free survival.

Supplementary Table 1 Quantity of extracted DNA using liquid biopsy. Pat. #, patient number, cfDNA, cell–free DNA in ng/µl, ctDNA, circulating tumor DNA in ng/ml; MAF, mutant allele frequency (%).
