# Supplementary figures and images for: Prediction of response to systemic treatment by kinetics of circulating tumor DNA in metastatic pancreatic cancer
Source: Front Oncol. 2022 Aug 30;12:902177. doi: 10.3389/fonc.2022.902177 (PMC9468369; doi:10.3389/fonc.2022.902177)

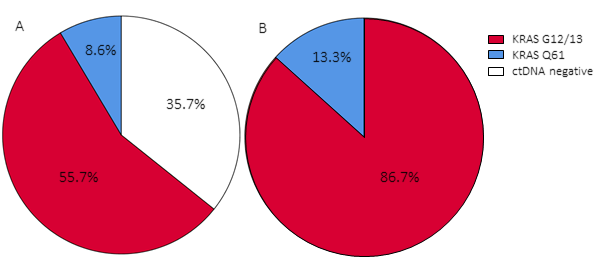

Supplement: Supplementary Figure 1 — Correlation of ctDNA and tumor burden. Correlation of MAF at baseline with total tumor volume (A), the dynamic change of MAF from baseline to restaging with the respective CA19–9 change (B) and similar relation for MAF change from baseline to 2 weeks after treatment initiation with the change of CA19–9 from baseline to restaging (C). ctDNA as indicator for tumor burden predicting response after 2 weeks of antineoplastic treatment (D). ctDNA, circulating tumor DNA; MAF, mutant allele frequency; nonPD, non–progressive disease; PD, progressive disease; R2, Spearman’s rho. [file Image_1.tif]

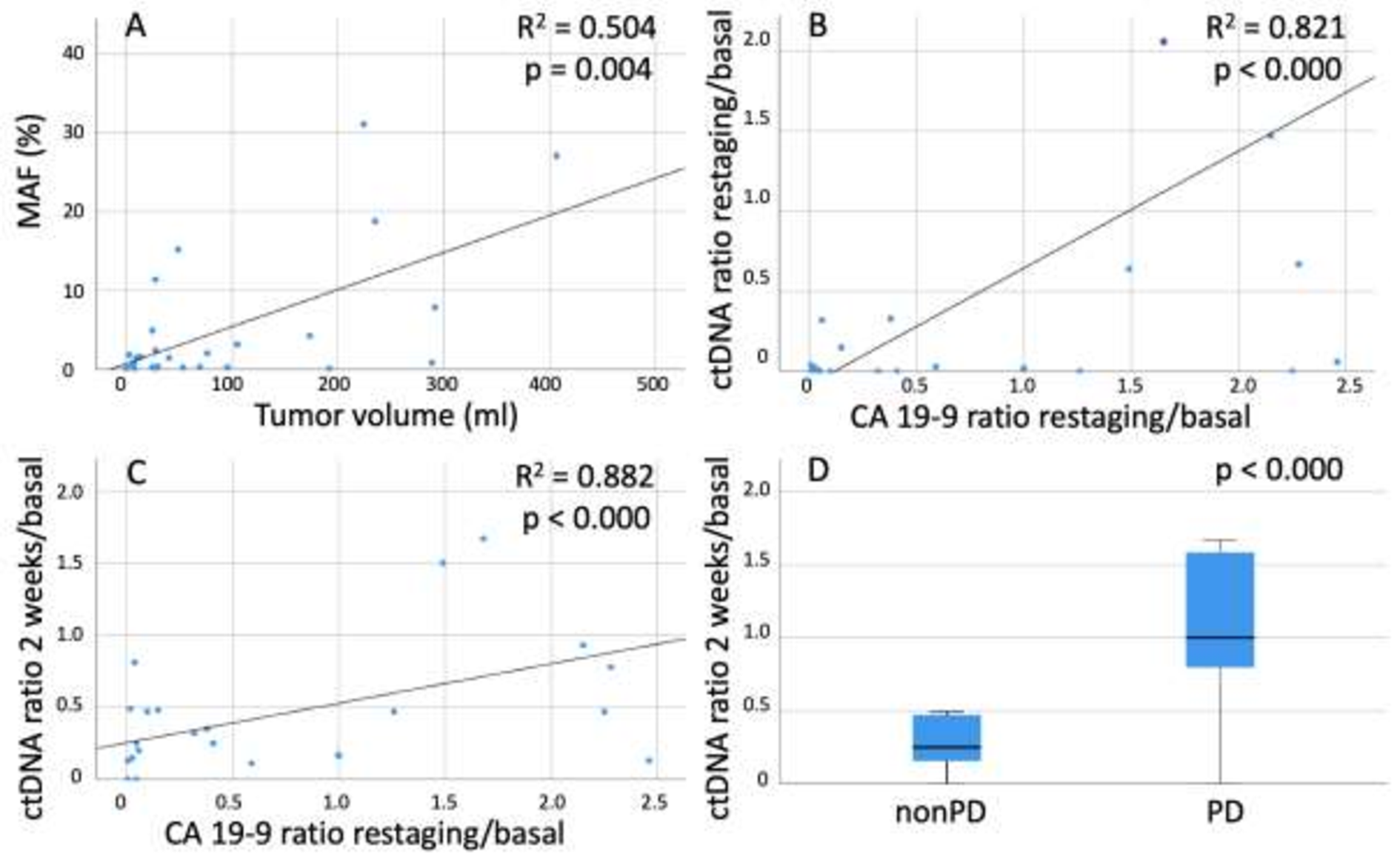

Supplement: Supplementary Figure 2 — Outcome of pretherapeutic ctDNA detection. Pretherapeutic ctDNA detectability correlates with worse OS (A, B) and PFS (C, D) in first line chemotherapy and regardless of treatment line. HR, hazard ratio; IQR, interquartile range; OS, overall survival; PFS, progression–free survival. [file Image_2.tiff]

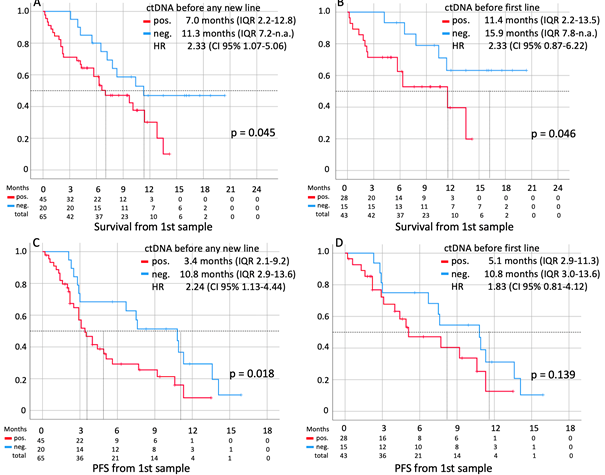

Supplement: Supplementary Figure 3 — Distribution of mutational pattern evaluating peripheral blood. [file Image_3.tif]

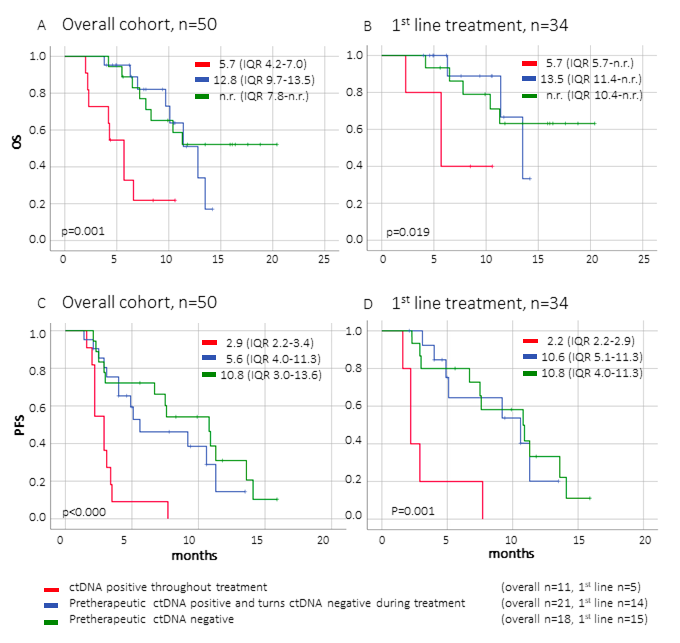

Supplement: Supplementary Figure 4 — Prognostic impact of ctDNA change during treatment. Patients with pretherapeutic positive ctDNA who turned ctDNA negative during palliative chemotherapy (blue) had significantly improved OS and PFS compared to patients whose ctDNA was positive throughout the course of therapy (red) in the overall cohort (A, C) and in patients receiving first line treatment (B, D). Furthermore, it seems that patients turning ctDNA negative during treatment show similar OS and PFS as patients with pretherapeutic negative ctDNA (green). ctDNA, circulating tumor DNA; IQR, interquartile range; OS, overall survival; PFS, progression–free survival. [file Image_4.tif]
