# Supplementary material for: Prediction of response to systemic treatment by kinetics of circulating tumor DNA in metastatic pancreatic cancer
Source: Front Oncol. 2022 Aug 30;12:902177. doi: 10.3389/fonc.2022.902177 (PMC9468369; doi:10.3389/fonc.2022.902177)
Supplement: Supplementary Table 1 — Quantity of extracted DNA using liquid biopsy. Pat. #, patient number, cfDNA, cell–free DNA in ng/µl, ctDNA, circulating tumor DNA in ng/ml; MAF, mutant allele frequency (%). [file Table_1.docx]

| **Quantity of extracted DNA using liquid biopsy**  **overview** | | | |
| --- | --- | --- | --- |
|  | cfDNA [ng/µl] | ctDNA [ng/ml] | MAF [%] |
| Pat. #1 | 1,35 | 6,70 | 0,25 |
| Pat. #2 | 0,54 |  |  |
| Pat. #3 | 11,70 | 741,78 | 3,17 |
| Pat. #4 | 0,90 | 7,20 | 0,40 |
| Pat. #5 | 0,99 | 16,04 | 0,81 |
| Pat. #6 | 1,92 | 10,37 | 0,27 |
| Pat. #7 | 0,90 | 26,46 | 1,47 |
| Pat. #8 | 191,00 |  |  |
| Pat. #9 | 0,74 |  |  |
| Pat. #10 | 6,28 | 12,56 | 0,10 |
| Pat. #11 | 1,41 | 42,02 | 1,49 |
| Pat. #12 | 0,97 | 31,62 | 1,63 |
| Pat. #13 | 0,74 |  |  |
| Pat. #14 | 0,88 |  |  |
| Pat. #15 | 0,88 | 138,69 | 7,88 |
| Pat. #16 | 0,84 |  |  |
| Pat. #17 | 0,81 | 7,34 | 0,45 |
| Pat. #18 | 0,75 | 27,60 | 1,84 |
| Pat. #19 | 0,46 |  |  |
| Pat. #20 | 1,92 |  |  |
| Pat. #21 | 0,45 | 2,80 | 0,31 |
| Pat. #22 | 1,63 |  |  |
| Pat. #23 | 0,63 | 3,53 | 0,28 |
| Pat. #24 | 1,00 |  |  |
| Pat. #25 | 0,48 | 1,82 | 0,19 |
| Pat. #26 | 8,32 | 3128,32 | 18,80 |
| Pat. #27 | 1,08 |  |  |
| Pat. #28 | 0,45 |  |  |
| Pat. #29 | 0,47 |  |  |
| Pat. #30 | 0,49 |  |  |
| Pat. #31 | 1,77 |  |  |
| Pat. #32 | 0,42 | 12,68 | 1,51 |
| Pat. #33 | 0,63 |  |  |
| Pat. #34 | 17,50 | 9485,00 | 27,10 |
| Pat. #35 | 4,80 | 1459,20 | 15,20 |
| Pat. #36 | 0,66 |  |  |
| Pat. #37 | 0,97 | 40,35 | 2,08 |
| Pat. #38 | 1,68 |  |  |
| Pat. #39 | 11,40 | 2599,20 | 11,40 |
| Pat. #40 | 31,70 | 19717,40 | 31,10 |
| Pat. #41 | 5,19 | 442,19 | 4,26 |
| Pat. #42 | 1,52 |  |  |
| Pat. #43 | 1,05 | 3,15 | 0,15 |
| Pat. #44 | 0,58 | 1,08 | 0,09 |
| Pat. #45 | 0,60 | 59,40 | 4,95 |
| Pat. #46 | 1,32 | 3,96 | 0,15 |
| Pat. #47 | 2,42 | 40,90 | 0,85 |
| Pat. #48 | 0,88 | 42,59 | 2,42 |
| Pat. #49 | 7,00 | 4060,00 | 29,00 |
| Pat. #50 | 0,70 |  |  |
| Pat. #51 | 1,25 | 2,50 | 0,10 |
| Pat. #52 | 0,32 | 0,64 | 0,10 |
| Pat. #53 | 0,82 | 138,91 | 8,47 |
| Pat. #54 | 0,65 | 88,40 | 6,80 |
| Pat. #55 | 0,25 | 18,85 | 3,77 |
| Pat. #56 | 33,30 |  |  |
| Pat. #57 | 1,17 |  |  |
| Pat. #58 | 1,81 | 124,17 | 3,43 |
| Pat. #59 | 1,09 | 112,92 | 5,18 |
| Pat. #60 | 0,95 | 1,90 | 0,10 |
| Pat. #61 | 1,27 | 3,81 | 0,15 |
| Pat. #62 | 0,20 |  |  |
| Pat. #63 | 0,73 | 44,53 | 3,05 |
| Pat. #64 | 1,79 | 397,38 | 11,10 |
| Pat. #65 | 5,80 |  |  |
| Pat. #66 | 2,16 | 146,02 | 3,38 |
| Pat. #67 | 0,82 |  |  |
| Pat. #68 | 1,66 | 19,46 | 0,59 |
| Pat. #69 | 1,24 | 28,02 | 1,13 |
| Pat. #70 | 1,86 | 101,56 | 2,73 |

**Supplementary Table 1: Quantity of extracted DNA using liquid biopsy.**

Abbreviations: Pat. #: patient number, cfDNA: cell-free DNA in ng/µl, ctDNA: circulating tumor DNA in ng/ml, MAF: mutant allele frequency (%).
